# Legislation: Solar Tax Credit Loses Energy

**DOI:** 10.1289/ehp.116-a380a

**Published:** 2008-09

**Authors:** Charles W. Schmidt

The solar investment tax credit (ITC), created by the Energy Policy Act of 2005, allows those who invest in solar technology to deduct 30% of the purchase and installation costs incurred during the year from their taxable income. Although it’s been a boon for home and small business owners who can quickly install photovoltaic (PV) solar panels, water heaters, and other technologies, the ITC hasn’t provided comparable benefits to much larger solar power plants that could supply grid electricity to homes and businesses throughout the United States—a state of affairs that may be prolonged by political bickering.

In a technology known as concentrating solar power (CSP), arrays of reflective mirrors spread over hundreds of acres collect and focus the sun’s heat to make steam that, in turn, creates electricity by running a turbine engine. A total of 10 CSP plants built in sun-drenched regions of California and Nevada during the 1980s—plus a 1-MW demonstration plant in Arizona—already power more than 300,000 homes at a cost of roughly 15¢ per kWh, comparable to standard rates in many U.S. locations. Electric utilities in those states, and also in Florida and Hawaii, have agreed to purchase 4,500 MW of additional solar-generated electricity from a total of 16 planned CSP facilities, which could power up to 3.5 million American homes.

But before investors will fund those projects, they want to be sure ITC tax breaks will be available throughout plant construction. A typical CSP plant takes 4–6 years to build, explains Nate Blair, a senior energy analyst with the Department of Energy National Renewable Energy Laboratory. Congressional officials have proposed eight-year extensions to the ITC that would boost the entire solar industry while also making CSP costs more predictable in the long term. Those proposals are contained in two bills that are currently stalled in the Congress: HR 6049, which has been passed by the House of Representatives but not the Senate, and the Senate’s counterpart, S 3335, which has yet to be passed by either legislative chamber.

According to Carol Werner, executive director of the Environmental and Energy Study Institute, a Washington, DC, think tank, investors need to be assured the ITC will be available for each year of construction so they can negotiate prices in advance for the power they sell. Until an extension long enough to cover construction time-lines emerges, she says, these projects will remain in limbo.

“This is a big deal [because it] affects manufacturers’ and installers’ capacity decisions,” adds Justin Baca, a senior research analyst with the Solar Energy Industries Association (SEIA), a trade group. “An eight-year extension of the ITC . . . [is] much more effective than a series of short-term renewals.”

Working with congressional supporters on both sides of the aisle, the solar industry has tried since 2006 to pass an eight-year ITC extension. Monique Hanis, director of communications for the SEIA, says Congress still can’t agree on how to cover the approximately $1.5 billion cost—which is far less than the roughly $40 billion in annual tax credits passed on to the fossil fuel industry (including coal, natural gas, and oil) every year. “Hopefully the Senate will pass one of the two bills later in the fall,” Hanis says. Meanwhile, the ITC is set to expire at the end of 2008.

## Figures and Tables

**Figure:**
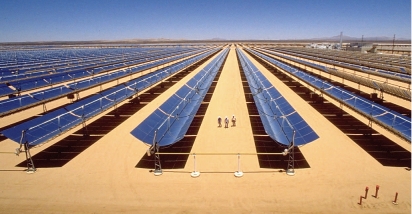
Nevada Solar One CSP plant Boulder City, Nevada

